# Supplementary material for: Image-based machine learning models for customized soil moisture management
Source: PLoS One. 2026 Feb 17;21(2):e0341904. doi: 10.1371/journal.pone.0341904 (PMC12912613; doi:10.1371/journal.pone.0341904)
Supplement: Attachment — Submitted filename: Soil_Image_1stRound_20250818.pdf [file pone.0341904.s003.pdf]

Manuscript Reference: PONE-D-25-27146
Title: Image-Based Machine Learning Models for Customized Soil Moisture Management
Journal: PLOS One

Reviewer #1

We would like to say thank you for your valuable comments to improve our paper. We have addressed the comments as explained below.

[1] Please add relevant references between lines 19 and 26.

Thank you for your comment. As suggested, we have added relevant references between lines 19 and 26 to strengthen the background and support the statements. The revised text now includes recent studies on smart farming and water resource management (Parra-López et al., 2025; Neophytides et al., 2024; Ingrao et al., 2023; Al-Kaisi & Broner, 2014; Kelly et al., 2025; Bounajra et al., 2024).

In manuscript:

(Line 19-26)

In the evolution of smart farming, water resource management technology has gained significant attention (Parra-López et al., 2025; Neophytides et al., 2024). As climate change intensifies water scarcity, efficient water resource management in agriculture has become an increasingly critical challenge (Ingrao et al., 2023). Moisture management in crop cultivation is a key factor in determining productivity and quality (Al-Kaisi & Broner, 2014). For example, inadequate moisture management can cause direct economic losses through reduced plant growth, increased pest and disease occurrence, and decreased yields (Ingrao et al., 2023). Precise moisture management requires accurate understanding crop water requirements based on growth stages and environmental conditions, and then providing the appropriate amount of water for each plant at the right time (Kelly et al., 2025; Al-Kaisi &Broner, 2014; Bounajra et al., 2024).

[2] Similarly, which studies are referenced in line 43? Please review the Introduction section to ensure all citations are properly included.

We have revised the citation for line 43 to ensure accuracy, and the sentence now cites European Parliamentary Research Service (2023), Akkem et al. (2023), and Soussi et al. (2023) as the relevant studies. In addition, as suggested, we reviewed the entire Introduction section to ensure the section is supported by relevant literature, and we have added citations where necessary.

In manuscript:

(Line 43)

Most artificial intelligence research related to smart farms incorporating various digital technologies has primarily focused on uniform management at the field or greenhouse level. Limited studies exist on customized cultivation management systems that consider the growth status of individual crops (European Parliamentary Research Service, 2023; Akkem et al., 2023; Soussi et al., 2023).

[3] The content between lines 72 and 82 appears to be background information. I suggest synthesizing this content and incorporating it into the Introduction.

Thank you for your comment. We carefully reviewed the content between lines 72 and 82 and agree that it provides important background information on the target crop. While your suggestion was to incorporate this content into the Introduction, we determined that it is more closely related to the description of experimental materials and design. Therefore, in revising the manuscript, we reorganized Section 2.1 Experimental Setup for Customized Growth Management to improve clarity and logical flow, creating two subsections: 2.1.1 Target Crop: Wild-simulated Ginseng and 2.1.2 Experiment Setup. The content originally found between lines 72 and 82 has been moved to the Target Crop subsection, where it directly supports the experimental design.

Below is the revised text for 2.1.1 Target Crop: Wild-simulated Ginseng, extracted from the reorganized Section 2.1 Experimental Setup for Customized Growth Management. Only the relevant subsection is presented here for clarity.

In manuscript:

(Line 79-92)

2.1.1. Target Crop: Wild-simulated Ginseng

High value-added agricultural and forest products command premium prices relative to their yield, highlighting the need for customized management to maximize productivity. The market price per gram of wild-simulated ginseng (WSG) exceeds that of cultivated ginseng by more than 20-fold, highlighting its prominence as a high-value forest product (Han et al., 2017; Jeong et al., 2019). Most WSG is marketed after more than seven years of cultivation. The quality and price are determined by various factors such as region, shape, weight, and other attributes that affect market value (Moon et al., 2019; Park et al., 2013; Shaoqing et al., 2013). Since the survival rate of WSG decreases dramatically from the fourth year onward, effective management strategies are required to improve survival rates and produce high-quality ginseng (Korea Forest Service, 2019; Woo, 2016). To establish legal standards for WSG and clearly distinguish it from cultivated ginseng, we investigated changes in WSG characteristics after a short post-treatment period following pre-germination transplantation. Accordingly, five-year-old WSG cultivated in Pyeongchang, Gangwon Province, was selected and transplanted into experimental soil as the target forest product for this study.

[4] In lines 104–105, is the soil moisture reported on a gravimetric or volumetric basis? Please clarify this point.

Thank you for your comment. The soil moisture content (50 ± 10%) reported in lines 104–105 was expressed as a percentage of the fully saturated condition of the soil, based on two-point calibration (0% for oven-dry soil and 100% for saturation). While the sensor output was calibrated using volumetric water content readings from a pre-calibrated device, the reported value represents the relative saturation level rather than an absolute volumetric water content. We have clarified this distinction in the revised manuscript.

In manuscript:

(Line 112-116)

Following transplantation, the soil moisture content was adjusted to 50 ± 10% of saturation, based on two-point calibration (oven-dry and fully saturated conditions), to provide a suitable starting environment. In this study, soil moisture content refers to the relative saturation of the soil, expressed as a percentage with 0% for oven-dry soil and 100% for saturation. Hereafter, this is referred to simply as soil moisture content.

[5] In Section 2.2, the authors describe the soil moisture monitoring system. Arduino-based soil moisture sensors are known for their limited accuracy (see for example https://doi.org/10.3390/s23052451), which can harm actual soil water conditions.

Additionally, the manuscript mentions that only two points were used for sensor calibration. How can the authors ensure the reliability of the sensors using only a two-point calibration approach for Arduino-based sensors?

Thank you for your comment and for highlighting the potential limitations of Arduino-based soil moisture sensors. We acknowledge that such sensors, in general, have lower absolute accuracy compared to high-grade instruments. In our study, to minimize this limitation, we individually calibrated each Arduino-based sensor against a pre-calibrated professional soil moisture meter prior to the experiment. The professional device had undergone factory calibration, ensuring a reliable reference for the calibration process.

Although a high-grade instrument was available, we opted to use Arduino-based sensors because our experimental design required simultaneous real-time monitoring of soil moisture across multiple pots. This was not feasible with a single high-grade device. Among the various types of Arduino-compatible sensors, we selected capacitive sensors, which are known to be more resistant to corrosion than resistive types. Furthermore, the experiment was conducted over a relatively short period, reducing the risk of sensor degradation. These measures, combined with controlled environmental conditions, allowed us to maintain sufficient reliability for our experimental objectives while addressing the known limitations of Arduino based sensors.

[6] In line 130, the authors mention a "100%" volumetric water content. Would this not refer to the degree of saturation of the soil sample rather than its volumetric water content? Please verify and ensure that the correct concepts and terminology are used consistently throughout the manuscript.

Thank you for your comment. As clarified in our response to Comment [4] and reflected in the revised Section 2.1, we defined "soil moisture content" based on a two-point calibration (0% for oven-dry soil and 100% for saturation). Therefore, the value referred to as "100%" in the original text indicates full saturation rather than volumetric water content in the strict sense. To maintain consistency, we have revised the manuscript to use the term "soil moisture content" throughout, which hereafter denotes relative saturation as defined in Section 2.1.

In manuscript:

(Line 140-141)

Based on two-point calibration, the average sensor output was 542 ± 54 at 0% (oven-dry) and 344 ± 50 at 100% (saturation).

[7] The content between lines 239 and 246 also appears to be background information. Please remove it from Section 2.4.2 and integrate a synthesized version into the Introduction.

Thank you for your helpful comment. In accordance with your suggestion, we have removed the background description from Section lines 239–246 in the original manuscript and integrated the relevant content into the Introduction to improve the manuscript.

In manuscript:

(Line 30-33)

Since each soil layer plays a distinct role in crop growth, understanding soil moisture variation by depth is also essential (He et al., 2021). The upper layer reflects rapid irrigation and evaporation changes (USDA, 1991), the middle layer is the primary zone for root activity, and the bottom layer functions as a reservoir that buffers excess moisture (Bai et al., 2025; He et al., 2025).

[8] In lines 424–426, could the reduced reliability of the soil moisture data have influenced the findings? Please consider and address this possibility.

Thank you for the comment. In the section referenced (lines 424–426), the increased prediction uncertainty at high-moisture conditions arises primarily from image-domain factors rather than degraded label reliability. Near saturation, the soil surface becomes darker and more homogeneous, pools of water introduce specular reflections, and the peatmoss/perlite mixture reduces micro-texture contrast, collectively weakening the visual cues available to the model. By design, ground-truth moisture labels were obtained from short-term, individually calibrated sensors and data were collected before canopy occlusion, which limited label noise. We have added a note to clarify that the observed uncertainty is mainly due to diminished discriminability in surface imagery under high moisture, while label reliability was managed through calibration and controlled acquisition.

In manuscript:

(Line 431-436)

Across all models, there was a general trend of increased prediction uncertainty under high-moisture conditions, which is likely due to the subtle changes in the soil texture that complicate accurate prediction. The findings highlight that an effective feature extraction strategy plays a more critical role in predictive performance than model complexity alone. Notably, the

higher uncertainty under near-saturation is attributable to reduced visual discriminability of the soil surface (darkening, specular reflections, and homogenization of the peatmoss/perlite mixture), which limits image-based cues.

[9] In lines 435–436, the authors seem to be referring again to the degree of saturation. If so, please correct the terminology.

We appreciate the reviewer's comment regarding the terminology. To address this, we clarified in the Methods section that soil moisture content in this study is defined as relative saturation (%) based on two-point calibration (oven-dry and fully saturated). We also revised Section 4.2.1 to explicitly state that the reported values represent relative saturation.

In manuscript:

(Line 444-445)

The bottom layer showed the highest mean soil moisture content, measured as relative saturation, with a mean value of 65.33% and a standard deviation of 15.01%.

[10] Currently, the Discussion section summarizes the main findings and highlights the study's limitations and contributions. However, a typical Discussion should also compare the results with findings from other studies. I strongly recommend revising this section to include comparisons with relevant recent literature.

We thank you for this important suggestion. In the revised manuscript, we have substantially expanded the Discussion to include explicit comparisons with recent literature. Specifically, we now contrast our image–sensor paired approach with prior sensor-based methods that required dense sensor networks for subsurface monitoring (Lloret et al., 2021). We also compare our plant-scale results with recent remote sensing studies that used satellite or UAV imagery for soil moisture estimation at larger scales (Peng et al., 2024; Lamichhane et al., 2025; Hernandez et al., 2025). Furthermore, we discuss similarities with imaging studies that employed hyperspectral and phenotyping platforms for assessing canopy water status (Furbank & Tester, 2011; Ge et al., 2018). By situating our findings within these recent advances, we highlight the novelty of our approach namely, demonstrating that simple RGB imagery can enable robust, plant-level moisture estimation with reduced sensor dependency. These revisions strengthen the Discussion by clarifying how our study complements and extends existing research.

Reviewer #2

We would like to say thank you for your valuable comments to improve our paper. We have addressed the comments as explained below.

[1] Maintain consistent use of the Oxford comma throughout lists.

Thank you for your observation. In the revised manuscript, we carefully reviewed all lists and ensured the consistent application of the Oxford comma. For example, the list of models in the Results section was revised to read "ResNet50, EfficientNetB0, MobileNetV2, InceptionV3, DenseNet121, and NASNetMobile." This consistent usage enhances readability and maintains stylistic uniformity throughout the manuscript.

[2] Replace passive voice were unnecessary, especially in methodology sections, with active constructions for clarity.

We appreciate your suggestion. We revised the manuscript to reduce unnecessary passive voice, particularly in the Methodology and Results sections. Whenever possible, sentences were rewritten in active voice to improve clarity and readability. For example, the previous phrasing "The number of trees was adjusted and evaluated" was revised to "We adjusted and evaluated the number of trees." These revisions highlight the authors' role in the research process and improve narrative flow.

[3] Avoid stacking multiple clauses in one sentence; break them into two where necessary.

We agree with your comment. Throughout the manuscript, sentences that contained multiple clauses were revised and divided into two or more concise statements to enhance readability. For instance, in the Results section, the sentence "As C increased beyond 10, performance declined, with the R^2 dropping to 73.9% at C = 1000" was revised into "As C increased beyond 10, performance declined. The R^2 dropped to 73.9% at C = 1000." These changes improve clarity and ensure that the manuscript is easier to follow for readers.

[4] Use consistent units of measurement (e.g., always clarify if % is gravimetric or volumetric moisture content).

Thank you for raising this important point. We have revised the manuscript to clarify that soil moisture content is expressed as relative saturation, where 0 percent corresponds to oven-dry

soil and 100 percent corresponds to fully saturated soil. To avoid ambiguity, we now consistently use this definition throughout the text and explicitly state that the values refer to relative saturation rather than gravimetric or volumetric content.

[5] Use either American or British English consistently (e.g., "analyze" vs. "analyse").

Thank you for pointing this out. The manuscript has been carefully revised to ensure consistent use of American English spelling and style throughout the text.

Reviewer #3

We would like to say thank you for your valuable comments to improve our paper. We have addressed the comments as explained below.

[1] I recommend adding a timeseries figure including both actual and predicted soil moisture (of Figure 4 and Figure 5). This allows for further understanding of the capabilities and limitations of the image-based estimation. If this is not possible, please explain why not?!

We sincerely appreciate your constructive suggestion to include a time-series plot comparing actual and predicted soil moisture. We fully agree that such a visualization could provide additional insights into the temporal dynamics of prediction models. However, our study was specifically designed to evaluate the accuracy of image-based models for estimating soil moisture at given observation times, rather than monitoring its temporal evolution.

The dataset was collected as independent samples measured at distinct time points under varying conditions, rather than as continuous monitoring data. Consequently, constructing a meaningful time-series plot is not feasible because the data do not represent sequential changes in soil moisture. Instead, scatter plots comparing actual versus predicted values (Figures 4 and 5) were employed, as these are widely accepted and appropriate for assessing point-based prediction performance.

While a time-series analysis is beyond the scope of the present work, we acknowledge its potential value and agree that it would provide additional insights into the strengths and

limitations of image-based estimation. We thank you for this valuable suggestion, which will serve as a useful reference for guiding future research directions.

[2] What is the coefficient of determination between soil moisture at the upper and middle depths against the bottom layer? This check is important to understand the added value of the regression models used to predict the bottom layer.

Thank you for the insightful comment. Before developing the proposed models, we evaluated simple OLS baselines to predict bottom-layer soil moisture from upper and middle layers. The R^2 values were 0.349 (Top→Bottom), 0.503 (Middle→Bottom), indicating only moderate explanatory power. In contrast, our nonlinear tree-based models achieved test R^2 of 0.906 (Random Forest) and 0.903 (Bagging), demonstrating clear added value beyond simple depth-wise correlations. We have reflected this point qualitatively in the Results section to maintain clarity and focus in the manuscript.

In manuscript:

(Line 471-473)

Simple linear regression using soil moisture at the upper or middle depths exhibited only limited explanatory power for the bottom layer, thereby underscoring the need for more advanced nonlinear approaches.

[3] The discussion of the manuscript can be further strengthened. Here are some points that are worth discussing:

We sincerely thank the reviewer for the insightful and constructive comments, which have greatly improved the quality and clarity of our manuscript. In the revised version, we carefully considered each point raised and made corresponding modifications in the Discussion section as detailed below.

- The study focused on the seedling stage, which raises the question of robustness and generalizability of your approach to the different growth stages. For example, a shadow will be introduced as the growth progresses, suggesting an updated model training, thus reducing its practicality.

Regarding your concern that the study focused only on the seedling stage, we clarified in Section 5.1 that this design choice was intentional. At later growth stages, foliage naturally

occludes the soil surface, rendering image-based observation of soil moisture infeasible. By focusing on the seedling stage, we aimed to provide actionable insights for irrigation management during early development, which is a particularly critical and vulnerable phase for wild-simulated ginseng. At the same time, we acknowledged this limitation and explicitly noted that future research should extend the analysis to later growth stages for improved generalizability.

In manuscript:

(Line 541-554)

`

To better understand the unique growth environments and moisture requirements of individual non-timber forest products, this study developed a non-invasive, customized soil moisture management model by integrating image-based machine learning with sensor data. The approach was designed to overcome limitations of conventional smart farming systems that rely on uniform control based on average environmental conditions. These systems often overlook the unique growth needs of individual plants. Although sensor-based systems have advanced environmental monitoring capabilities, they are typically applied in a generalized manner across entire cultivation areas, making it difficult to reflect plant-level variability. To address these limitations, this study proposes an alternative approach that enables customized management by predicting soil moisture using only surface images, thereby minimizing the need for dense sensor deployment. The study was conducted at the seedling stage of wild-simulated ginseng, where soil remains visually accessible before being covered by foliage. While this stage-specific focus limits generalization across the entire growth cycle, it nonetheless underscores a practical contribution, as irrigation management is particularly critical during early development.

- The approach applied looks infeasible for the intended purpose of crop management on a large scale. A key question would be how such an approach can be feasibly implemented for large-scale areas.

We appreciate the reviewer’s concern regarding the scalability of the proposed approach. As clarified in the revised manuscript, the primary aim of this study was to demonstrate the feasibility of plant-level soil moisture estimation using the most accessible imaging device like smartphones. This design choice ensures practical applicability in smallholder contexts

and for high-value crops where individualized management is particularly important. However, we agree that direct extension of this approach to large-scale crop systems is limited. To address this, we have added a discussion in Section 5.3 and 5.4, highlighting that future research should explore integration with UAV platforms, distributed low-cost camera networks, or hybrid imaging–sensor frameworks. This clarification emphasizes that while our method is not intended as a standalone solution for large-scale monitoring, it contributes an essential foundation for scalable precision agriculture systems.

In manuscript:

(Line 574-597)

5.3. Practical contributions and agricultural implications

Beyond moisture estimation, the core contribution of this research lies in enabling plant-level management strategies based on non-destructive image data. Traditional cultivation practices often apply uniform treatments across crops, overlooking microenvironmental variation and individual physiological differences. Previous plant-based irrigation studies have emphasized the importance of individualized management (Jones, 2004), but practical methods have remained limited due to sensing costs and scalability issues. Our approach addresses this gap by allowing image-based assessment of plant-specific water status, supporting adaptive decisions tailored to each plant's developmental stage and soil environment.

Similar to recent imaging studies that evaluated leaf or canopy water status using hyperspectral and phenotyping platforms (Furbank & Tester, 2011; Ge et al., 2018), our framework demonstrates that even simple RGB imagery can provide actionable insights at the plant scale. Rather than merely improving irrigation efficiency, our approach promotes customized crop care, contributing to sustainable and resource-efficient cultivation. This aligns with the broader objectives of climate-smart agriculture (Lipper et al., 2014) by offering an accessible, sensor-free alternative for precision monitoring, particularly valuable in areas with limited infrastructure or water availability. Moreover, this system is especially suitable for managing high-value or sensitive crops, where minimal physical disturbance is essential. In future applications, the image-based approach shows potential for broader implementation. In this study, we primarily employed smartphone-based imaging to ensure accessibility for farmers, as smartphones are the most readily available and easy-to-use cameras in practice. This choice highlights the feasibility of applying our method for plant-level precision management in smallholder or high-value crop settings. However, direct scale-up to large-scale farming systems presents challenges, as smartphone-based imaging is not

practical for monitoring extensive areas. To address this limitation, future implementations may require integration with UAV platforms, distributed camera networks, or hybrid approaches combining imaging with sensor-based monitoring. Thus, while our current framework demonstrates clear value at the individual plant level, particularly for sensitive or high-value crops, its large-scale application will necessitate complementary technologies.

- Since the initial soil moisture was controlled (40-60%), how would changes in the initial soil moisture impact the performance of the AI model? Try to discuss this point and explain what is needed to quantitatively analyze this sensitivity.

We sincerely appreciate your thoughtful comment. The reported initial soil moisture (50 ± 10%) reflected the intrinsic property of the purchased soil and was adjusted only to provide a uniform starting condition following transplantation. Importantly, this initial setting did not constrain the training data, as soil moisture varied substantially during the experiment due to continuous irrigation and natural drying. Consequently, the image dataset captured a wide spectrum of soil moisture levels beyond the initial range, ensuring that the model learned from diverse conditions rather than being biased toward a fixed starting point. Nevertheless, we agree that explicitly evaluating sensitivity to initial soil moisture would further strengthen the generalizability of the framework, and we have clarified this point in the revised manuscript

In manuscript:

(Line 619-624)

Soil moisture at transplantation was adjusted to approximately 40–60% of saturation to provide a uniform starting condition, and subsequent irrigation and natural drying generated substantial variation throughout the experiment. The dataset therefore included a wide spectrum of soil moisture levels, allowing the model to learn from heterogeneous conditions rather than being confined to a narrow range. Nevertheless, systematic evaluation under more extreme or fluctuating regimes, as encountered in field environments, represents an important avenue for future research to strengthen robustness and generalizability.

- What was the rationale behind selecting 224x224 pixels? What is the implication of such a decision?

We appreciate the reviewer’s insightful comment. The decision to use 224×224 pixels was primarily based on two considerations. we employed convolutional neural network

architectures such as DenseNet that are widely pre-trained on ImageNet, where the standard input size is 224×224. Adopting this convention ensured architectural compatibility and allowed us to leverage transfer learning efficiently. Second, resizing all images to a uniform dimension was necessary to standardize inputs across samples and facilitate stable training.

We acknowledge that the choice of resolution may affect the granularity of visual information captured. While 224×224 provides a balance between computational efficiency and feature preservation, higher or lower resolutions could alter predictive robustness. Exploring this trade-off systematically is an important future direction, as different resolutions may be more optimal depending on crop type, imaging setup, or computational resources. We have now clarified this rationale and its implications in the revised manuscript.

In manuscript:

(Line 621-626)

Resolution also constitutes a limitation, as training images were standardized to 224×224 pixels. While this choice ensured compatibility with convolutional neural network architectures and facilitated efficient model training, the trade-offs among image resolution, computational cost, and predictive robustness remain insufficiently explored. Future studies should systematically examine these trade-offs, as different resolutions may prove more effective depending on crop type, imaging conditions, and computational resources.

- The practicality does not fully convince me of your second part (i.e., soil moisture estimation at deep layers from the upper and middle measurements). In which cases can such a model be useful? The rationale behind this analysis requires strong arguments to accept it.

We sincerely appreciate your valuable comment regarding the practicality of estimating deeper-layer soil moisture. Our long-term objective is to develop a low-intrusion decision support system capable of estimating upper, middle, and deep soil moisture from a single surface observation. This approach is particularly useful in high-value or sensitive crops and seedling beds where multi-depth probe installation is constrained by cost, invasiveness, and maintenance requirements, and it is also valuable in large-scale applications such as greenhouses, orchards, and forest understory environments where scalable and minimally intrusive monitoring is essential. The rationale for this approach lies in the fact that vertical soil moisture states are physically coupled through infiltration and retention processes, such that surface appearance reflects near-surface water content and, indirectly, recent flux history.

By combining image-based features with physics-aware constraints and multi-depth calibration data, a model can potentially map surface signals to deeper profiles. In future work, we acknowledge the need for further validation through the acquisition of multi-depth datasets and stratified evaluation across soil types and irrigation regimes to fully demonstrate practical applicability. We have clarified this point in the revised manuscript (Section 5.4).

[4] Lines 43-45: an example of references is missing.

Thank you for pointing this out. We have added representative references to support the statement in Lines 43–45, including recent studies on large-scale soil moisture monitoring using satellite and UAV data (e.g., Roy et al., 2023; Terlizzi et al., 2023). These additions provide sources for the background statement and improve the consistency of citations in the Introduction.

In manuscript:

(Line 52-55)

Previous studies have mainly emphasized developing models that manage overall soil conditions or facility environments based on information from large-scale monitoring systems using satellite data or drones (Roy et al., 2023; Terlizzi et al., 2023; Elsayed et al., 2022).

[5] Figure 1: Add dimensions to the cultivation system with more elaboration on its different components.

We appreciate your suggestion to provide more detail about the cultivation system. We have revised Figure 1 to include the dimensions of the Genesis FarmBot cultivation bed (1.5 m × 3 m, height 0.5 m) and elaborated on its main component, the X-Y-Z gantry. This revision clarifies the physical scale and setup of the experimental system.

[6] Lines 171-172: add references to the pre-trained models.

Thank you for the suggestion. We agree with the reviewer's comment. We have now added the appropriate references for the six pre-trained deep learning models used in this study (ResNet50, EfficientNetB0, MobileNetV2, InceptionV3, DenseNet121, and NASNetMobile). These references have been included in the revised manuscript in the Methods section (Lines 184–187) and listed in the References section.

In manuscript:

(Line 184-187)

We adopted six pre-trained deep learning architectures—ResNet50 (He et al., 2016), EfficientNetB0 (Tan & Le, 2019), MobileNetV2 (Sandler et al., 2018), InceptionV3 (Szegedy et al., 2016), DenseNet121 (Huang et al., 2017), and NASNetMobile (Zoph et al., 2018).

[7] Lines 167, 407: Typo in figure numbering

We carefully reviewed the numbering of all figures and tables throughout the manuscript. The inconsistencies identified at lines 167 and 407 were corrected, and the figure numbering has now been revised consistently across the text, figures, and references.

[8] Table 3: caption is not complete. "is used to reduce…"

We appreciate your careful observation. The caption of Table 3 has been revised to correct the incomplete sentence and now provides a complete and clear description.

[9] Lines 336-338: give different numbers for each question. Moreover, the equation of MSE is missing, and the second equation is for NRMSE, not RMSE. Make the necessary changes!

We thank you for pointing out this issue. We have revised the manuscript by adding the missing formula for MSE and correcting the mislabeled RMSE equation, which was previously presented as NRMSE. In addition, we clarified the definitions of MAE, MSE, RMSE, and R^2 to ensure correctness and consistency throughout the manuscript.

[10] I found the heading and subheading titles are long. I suggest making shorter titles.

We appreciate the reviewer's observation. Honestly, we did not initially consider the titles to be too long, but we agree that shortening them makes the manuscript clearer and easier to follow. Accordingly, we revised several section and subsection titles to be more concise.

[11] Table 4 should be split into Tabl 4 and Figure 4. Moreover, I suggest adding a legend that links the difference in the MAE values and the bubble size.

We appreciate your suggestion. Following the comment, Table 4 has been split into Table 4 and Figure 4 to improve readability and clarity. In addition, we have added a legend in Figure 4 to explicitly link the bubble size to the generalization gap. We believe these revisions enhance the interpretability of the results.

[12] Table 6: The ranking of the models is not correct in the caption. Change it to "random forest and bagging decision tree regression … followed by gradient boosting".

We corrected the caption of Table 6 to state that random forest and bagging decision tree regression achieved the best predictive performance, followed by gradient boosting, which is consistent with the reported R^2 and RMSE.

In manuscript:

(Line 461-466)

Performance comparison of six machine learning regression models for soil moisture prediction. Reported R^2 and RMSE correspond to the best cross-validated hyperparameters. Random forest and bagging decision tree regression achieved the best predictive performance, followed by gradient boosting. Polynomial regression and KNN regression showed moderate performance, while SVR with an RBF kernel yielded the lowest. All models were evaluated on the same dataset containing multi-depth soil moisture measurements.

[13] Figure 5: Add more information about the right panels in the caption.

We thank you for the helpful comment. Following the suggestion, we have revised the caption of Figure 6 to provide more information about the right panel. The updated caption now specifies that the right panel shows a scatter plot of actual versus predicted soil moisture values on the testing data using the optimized hyperparameter. It also clarifies that the black dashed line represents the 1:1 reference, and that tighter clustering of points along this line reflects better predictive performance.

In manuscript:

(Line 491-495)

Figure 6. Performance evaluation and comparison of six machine learning models for soil moisture prediction. Left panels display R^2 scores across different hyperparameter values. Red shaded areas represent the standard deviation from k-fold cross-validation on the test set, and blue lines indicate training performance. Right panel is a scatter plot of actual vs. predicted soil moisture on the testing data using the optimized hyperparameter. The black dashed line indicates the 1:1 reference, and tighter clustering of points along this line reflects better predictive performance.

[14] Type in line 550, change “Furesearch” to “Future research”.

Thank you for pointing out this typo. We have corrected “Furesearch” to “Future research” in the revised manuscript.

[15] Line 552: future work should explore other predictors beyond the measured spectrum (i.e., indices that can indicate changes in soil moisture).

We thank the reviewer for this valuable suggestion. We have revised the Discussion section (Line 552) to explicitly include the exploration of indices that directly reflect changes in soil moisture as future predictors, in addition to other spectral data.

In manuscript:

(Line 570-576)

Future research should explore how to apply this method to diverse crop types and field environments and incorporate additional spectral data such as thermal or multispectral imagery to enhance prediction robustness. In addition, developing and testing indices that directly reflect changes in soil moisture could provide more sensitive predictors beyond raw spectral inputs. Furthermore, integrating this system with plant growth models could enhance its ability to support adaptive, plant-level crop management strategies throughout the entire growth cycle, enabling more context-aware decisions beyond irrigation alone.
