# Supplementary material for: Image-based machine learning models for customized soil moisture management
Source: PLoS One. 2026 Feb 17;21(2):e0341904. doi: 10.1371/journal.pone.0341904 (PMC12912613; doi:10.1371/journal.pone.0341904)
Supplement: Attachment — Submitted filename: Review Report for PONE.docx [file pone.0341904.s001.docx]

**Peer Review Report**

**Manuscript ID**: PONE-D-25-27146

**Title**: Image-Based Machine Learning Models for Customized Soil Moisture Management

**Reviewer**: Dr. Iftikhar Ahmed

**General Evaluation**

This manuscript presents a novel and commendable attempt to non-invasively estimate soil moisture using RGB images in conjunction with machine learning models. The study is conducted on *wild-simulated ginseng*, a moisture-sensitive crop and leverages deep learning and ensemble-based regression models to predict soil moisture content at various depths. From a soil science perspective, the integration of image-based analysis with sensor data for soil moisture profiling is both timely and relevant, especially in the context of climate-smart and precision agriculture.

**Major Concerns and Recommendations**

**1. Soil Type and Texture Details**:

- The manuscript does not provide sufficient detail on soil physical properties (e.g., texture, bulk density, field capacity), which are critical to understanding water retention behavior and image-based interpretation.
- **Recommendation:** Include a table or paragraph detailing the physico-chemical characteristics of the soil mix used (beyond stating peat moss: perlite 6:4). This is essential to interpret the soil moisture dynamics.

**2. Moisture Calibration Units**:

- The sensor data appears to be in arbitrary units (0–1023) converted via calibration, yet final reported moisture predictions are in percentages.
- **Recommendation:** Clarify the conversion method from raw sensor values to volumetric water content. Include calibration curves or equations if available.

**3. Soil Moisture Ranges**:

- The reported moisture levels (e.g., >60%) seem high for peat-perlite mixes, unless the value is gravimetric or saturated conditions were used.
- **Recommendation:** Confirm the unit (volumetric vs gravimetric) and measurement technique used for "moisture %" throughout the manuscript.

**Minor Suggestions**

**1. Terminology**: Terms like “bottom layer = 3 cm from base” may confuse readers, as this is typically referred to as "deep layer." Consider using conventional soil profile terminology (surface, subsoil, etc.).

**2. Figures and Tables**:

- Include soil moisture distribution profiles over time (e.g., after irrigation) to visualize how moisture moves through layers.
- A confusion matrix or regression scatterplots with residuals may further support the model’s predictive capability.

**3. Reference Updates**:

- Some references are cited as "2024/2025" which may still be in press or unavailable. Ensure all references are accessible and published.

**Recommendation**

**Minor Revision**

The manuscript is well-written, methodologically sound and offers significant contributions to both soil science and smart agriculture domains. With minor revisions mainly concerning soil property reporting and clarity in moisture measurement the study would be suitable for publication.
