# Supplementary material for: Image-based machine learning models for customized soil moisture management
Source: PLoS One. 2026 Feb 17;21(2):e0341904. doi: 10.1371/journal.pone.0341904 (PMC12912613; doi:10.1371/journal.pone.0341904)
Supplement: Attachment — Submitted filename: Revision_2ndRound.docx [file pone.0341904.s004.docx]

**Manuscript Reference:** PONE-D-25-27146

**Title:** Image-Based Machine Learning Models for Customized Soil Moisture Management

**Journal:** PLOS One

**Reviewer [2]**

We would like to say thank you for your valuable comments to improve our paper. We have addressed the comments as explained below.

**[1]** The manuscript does not provide sufficient detail on soil physical properties (e.g., texture, bulk density, field capacity), which are critical to understanding water retention behavior and image-based interpretation.

We appreciate your comment regarding the lack of detail on soil. In this study, all plants were grown in a commercially standardized peat moss and perlite mixture (6:4 ratio), which was intentionally kept constant across experimental units because the objective was to examine moisture stress responses and image-based predictions within a uniform substrate rather than to compare different soil types. For this reason, soil physical properties were not treated as independent experimental variables. However, we fully agree that the general physical characteristics of the substrate are important for understanding water retention behavior and sensor interpretation. To address this concern, we have revised the manuscript to include a description of the typical physical properties of peat–perlite mixtures based on manufacturer information and relevant literature (high porosity, low bulk density, and moderate-to-high water-holding capacity). We appreciate your helpful suggestion, which has strengthened the clarity and contextualization of the experimental setup.

In manuscript:

*(Line 97-106) WSG seedlings were grown under controlled greenhouse conditions following transplantation. The image-based analysis in this study focused on the early growth period after transplantation, a stage during which wild-simulated ginseng is highly sensitive to moisture conditions. Moisture stress during this period has a direct influence on seedling survival and early physiological responses, including changes in saponin composition[53]. To ensure consistent rooting conditions across plants, we used a standardized peat moss and perlite substrate in a 6:4 ratio, which is commonly adopted for ginseng seedling cultivation[54, 55]. This substrate composition is known to provide high porosity, low bulk density, and a favorable balance between aeration and water-holding capacity. Such physical characteristics create a uniform and supportive rooting environment, which is particularly important during the moisture-sensitive early growth stage of WSG*

**[2]** The sensor data appears to be in arbitrary units (0–1023) converted via calibration, yet final reported moisture predictions are in percentages.

Thank you for raising this point regarding the conversion of raw sensor outputs into soil moisture values. The capacitive sensors used in this study generate electrical signals in the 01023 range, which must be converted before meaningful interpretation is possible. To address this concern, we have clarified the calibration procedure in the revised manuscript. Specifically, we now explain that soil moisture (%) values were derived using a standard two-point calibration approach based on oven-dry (0%) and fully saturated (100%) reference states. This method converts the raw electrical signals into normalized moisture estimates rather than direct volumetric water content. We have updated the Methods section to explicitly describe this conversion process and to provide the corresponding calibration values. We believe these revisions improve transparency and strengthen the methodological clarity of the study. We sincerely appreciate your suggestion.

In manuscript:

*(Line 130-142) Soil moisture status was measured using capacitive moisture sensors integrated into the Arduino-based moisture management system. Each sensor was installed individually for each WSG plant to enable customized monitoring and control of growth conditions. To avoid signal distortion, which is commonly caused by corrosion in resistive sensors, we used HW-390 capacitive soil moisture sensors. The capacitive soil moisture sensors used in this study detect changes in dielectric permittivity associated with varying soil moisture levels based on frequency domain reflectometry (FDR). These readings are converted into electrical signals ranging from 0 to 1023. Higher values correspond to lower moisture conditions. To convert the raw sensor outputs into normalized moisture (%) values, we applied a two-point calibration procedure using oven-dry (0%) and fully saturated (100%) reference states. The average sensor readings were 542 ± 54 at 0% and 344 ± 50 at 100%. In this study, the moisture (%) values represent a normalized index derived from the calibrated sensor signals rather than direct volumetric or gravimetric water content.*

*For the soil-depth analysis experiment, we installed three moisture sensors per plant at distinct heights above the pot base: 15 cm (top layer), 10 cm (middle layer), and 3 cm (deep layer). These layer designations were adopted to align with conventional soil profile terminology and to provide clearer interpretation of the soil structure within the pot.*

**[3]** The reported moisture levels (e.g., >60%) seem high for peat-perlite mixes, unless the value is gravimetric or saturated conditions were used. Confirm the unit (volumetric vs gravimetric) and measurement technique used for "moisture %" throughout the manuscript.

We appreciate insightful comment regarding the interpretation of the moisture (%) values. We would like to clarify that the moisture percentages reported in this study do not represent direct volumetric or gravimetric water content. Instead, these values correspond to a normalized index derived from the calibrated electrical outputs of the capacitive sensors. The raw sensor signals (0–1023) were converted into normalized moisture (%) values using a two-point calibration procedure based on oven-dry (0%) and fully saturated (100%) reference conditions. As a result, values such as >60% indicate relative moisture levels with respect to saturation rather than absolute volumetric water content. To avoid any ambiguity, we have revised the Methods section to explicitly state that moisture (%) is presented as a normalized index rather than a physical water content measure. We thank you for this helpful suggestion, which has improved the clarity of the manuscript.

In manuscript:

*(Line 135-137) In this study, the moisture (%) values represent a normalized index derived from the calibrated sensor signals rather than direct volumetric or gravimetric water content.*

**[4]** Terms like “bottom layer = 3 cm from base” may confuse readers, as this is typically referred to as "deep layer." Consider using conventional soil profile terminology (surface, subsoil, etc.).

We sincerely appreciate your comment regarding the terminology used to describe the soil layers. As noted, the expression “bottom layer = 3 cm from base” may lead to confusion and does not align well with conventional soil profile terminology. In response, we have revised the manuscript to use clearer and more standard terms. Specifically, the sensors positioned 15 cm, 10 cm, and 3 cm above the pot base are now described as the top, middle, and deep soil layers, respectively. We believe this terminology provides a more intuitive understanding of the soil structure and improves the clarity of the experimental description. In addition, we have carefully reviewed the entire manuscript and updated all related terminology to ensure consistency throughout the text. We sincerely thank you for this helpful suggestion.

**[5]** Include soil moisture distribution profiles over time (e.g., after irrigation) to visualize how moisture moves through layers. A confusion matrix or regression scatterplots with residuals may further support the model’s predictive capability.

We sincerely appreciate your suggestions regarding the visualization of soil moisture dynamics and model performance. We would like to clarify that the present study did not track soil moisture redistribution over time after irrigation. Our experiment matched the soil moisture measured by sensors with the images captured at the same moment to train the models for estimating individual soil moisture conditions. Because continuous time-series data were not collected, we are unable to generate soil moisture distribution profiles over time. Nonetheless, we find your suggestion highly valuable and agree that incorporating temporal moisture redistribution analysis would be a meaningful direction for future advanced research.

Regarding your recommendation to include a confusion matrix or regression visualizations, we interpreted the suggestion for a confusion matrix as an interest in improving clarity in model evaluation. However, since the present study does not use a classification model, tools that are specifically designed for classification such as confusion matrices cannot be applied within our modeling framework. As for regression-based visualizations, scatterplots and residual distributions have already been provided in Figure 5 and Figure 6, which illustrate the predictive performance and residual patterns of the models. We appreciate your feedback and believe that the suggestions have helped strengthen the clarity of the manuscript.

**[6]** Some references are cited as "2024/2025" which may still be in press or unavailable. Ensure all references are accessible and published.

We appreciate your comment regarding the references listed as 2024/2025. We have thoroughly reviewed the reference list and corrected all instances of 2024/2025, ensuring that only accessible and fully published sources are cited in the revised manuscript. Thank you for pointing this out.

**Reviewer [3]**

We would like to say thank you for your valuable comments to improve our paper. We have addressed the comments as explained below.

**[1]** I noticed that my minor comment about Figure 2 was missed. Please add scale per subpanel.

We appreciate helpful suggestion regarding the inclusion of scale bars in Figure 2. Although the images were captured at a fixed camera distance, no physical reference object or measured scale was included during image acquisition. Under these conditions, it is not possible to derive an accurate physical scale, and adding an arbitrary scale bar could mislead readers by implying a level of spatial or dimensional accuracy that the images do not provide. Figure 2 is intended solely to offer a qualitative illustration of visual differences across moisture levels, rather than to support spatial or size-dependent interpretation. To avoid potential misunderstanding while maintaining clarity, we have added a note to the figure caption indicating that the images are not to scale.

In manuscript:

Figure 2 Sample soil surface images at different moisture levels. These images are provided solely for qualitative illustration, and no spatial or physical scale is implied (not to scale).

**[2]** I suggest adding the R2 values of the linear models for better clarification.

We sincerely appreciate your thoughtful comment regarding the inclusion of R² values. We would like to clarify that all R² values associated with the models used in this study are already fully reported in Table 4 and Table 6 of the revised manuscript. These values reflect the best-performing models obtained. However, in the event that we have misunderstood your intended meaning or if you were referring to a different form or context of R² beyond what has already been provided, we would be more than willing to incorporate any additional R² values or clarifications as necessary. We appreciate your careful evaluation and remain grateful for the feedback.

**[3]** Training a ML model to predict the deeper layer from the surface layer requires training hence information about the deeper soil moisture (from sensors?).

We sincerely appreciate the comment regarding the relationship between surface imaging and deeper-layer soil moisture estimation. To clarify the scope of the current study, the deeper-layer analysis was conducted using actual sensor measurements collected at 15 cm, 10 cm, and 3 cm above the pot base, and these data were used to train and evaluate the depth-specific prediction models. Therefore, the deeper-layer models were developed with the necessary information. At the same time, the present work does not implement an end-to-end system that predicts deeper soil moisture directly from surface images. The image-based component was designed only for surface-layer estimation, and integrating surface imagery with deeper-layer prediction remains a future research goal. We agree with the reviewer that achieving such integration would require careful consideration of soil heterogeneity and the challenges of transferring models across different conditions. We appreciate your insight, and we have revised the manuscript to better clarify the scope of the current framework and the implications for future development.

In manuscript:

*(Line 570-579) Although this study successfully developed depth-specific soil moisture models using sensor measurements collected at 15 cm, 10 cm, and 3 cm above the pot base, the surface-image–based component was applied only to the shallow layer. As a result, the present framework does not yet integrate surface imaging into an end-to-end system for predicting deeper soil moisture. Developing such an integrated approach remains a future goal but would require corresponding ground-truth data for deeper layers as well as methods capable of addressing the substantial spatial heterogeneity of soil moisture conditions. Previous studies have similarly reported that machine learning based soil moisture models often exhibit reduced performance when applied across different locations or soil conditions unless multi-site calibration or domain adaptation techniques are employed[77-80]. Future extensions of the framework will therefore require multi-environment datasets and modeling strategies that explicitly account for spatial heterogeneity in soil moisture dynamics.*
